# Self-Immolative Domino Dendrimers as Anticancer-Drug Delivery Systems: A Review

**DOI:** 10.3390/pharmaceutics16050668

**Published:** 2024-05-16

**Authors:** Karolina Kędra, Ewa Oledzka, Marcin Sobczak

**Affiliations:** 1 Institute of Physical Chemistry, Polish Academy of Sciences, 44/52 Kasprzaka Str., 01-224 Warsaw, Poland; kkedra@ichf.edu.pl; 2 Faculty of Pharmacy, Department of Pharmaceutical Chemistry and Biomaterials, Medical University of Warsaw, 1 Banacha Str., 02-097 Warsaw, Poland; eoledzka@wum.edu.pl

**Keywords:** biomedical dendrimers, self-immolative domino dendrimers, drug delivery systems, anti-cancer drug delivery systems, controlled drug release

## Abstract

Worldwide cancer statistics have indicated about 20 million new cancer cases and over 10 million deaths in 2022 (according to data from the International Agency for Research on Cancer). One of the leading cancer treatment strategies is chemotherapy, using innovative drug delivery systems (DDSs). Self-immolative domino dendrimers (SIDendr) for triggered anti-cancer drugs appear to be a promising type of DDSs. The present review provides an up-to-date survey on the contemporary advancements in the field of SIDendr-based anti-cancer drug delivery systems (SIDendr-ac-DDSs) through an exhaustive analysis of the discovery and application of these materials in improving the pharmacological effectiveness of both novel and old drugs. In addition, this article discusses the designing, chemical structure, and targeting techniques, as well as the properties, of several SIDendr-based DDSs. Approaches for this type of targeted DDSs for anti-cancer drug release under a range of stimuli are also explored.

## 1. Introduction

Effective and biosafe therapy for cancer is still a significant challenge for modern medicine and pharmacy. As is commonly known, the basis of cancer treatment is radiotherapy and surgical chemotherapy. Inhibiting the proliferation of cancer cells, disease progression, and metastases is the goal of systemic chemotherapy treatment. However, chemotherapeutics unfortunately cause dangerous side effects. As a result, one of the strategic directions for ensuring effective and safe chemotherapy is the search for innovative anti-cancer drug delivery systems (DDSs). There is an urgent need for new anti-cancer DDSs that sustain or enhance the effectiveness of chemotherapy while reducing the response’s stringency and adverse effects. Many polymeric biomaterials have been employed as delivery vehicles for drugs and imaging agents. These include micro- or nanoparticles, hydrogels, dendrimers, nanofibers, prodrugs, macromolecular conjugates, etc. A particularly interesting group of biomedical polymers are dendrimers. Dendrimers are hugely branched polymers composed of the following three diverse architecture constituents: core, branches, and terminal functional groups. The core constitutes the central part of the dendrimeric scaffold, which may be a molecule with at least two similar chemical functional groups. The branches are the repeat units of dendrimers, which begin from the core. The geometric repetition of the dendrimeric branches results in the formation of radially centric layers called “generations.” The terminal functional groups are stationed at the periphery of the dendrimeric scaffold and are the most crucial factor for determining the property of the dendrimers [1,2,3,4,5,6,7]. 

Self-immolative domino dendrimers (SIDendr) are one of the more exciting and prospective types of anti-cancer DDSs. SIDendr are a new class of molecules designed so that a cascade of intramolecular reactions occurs in response to the cleavage of the trigger moiety, resulting in molecular fragmentation and the release of many drug molecules [8,9,10,11]. 

**Figure 1:**
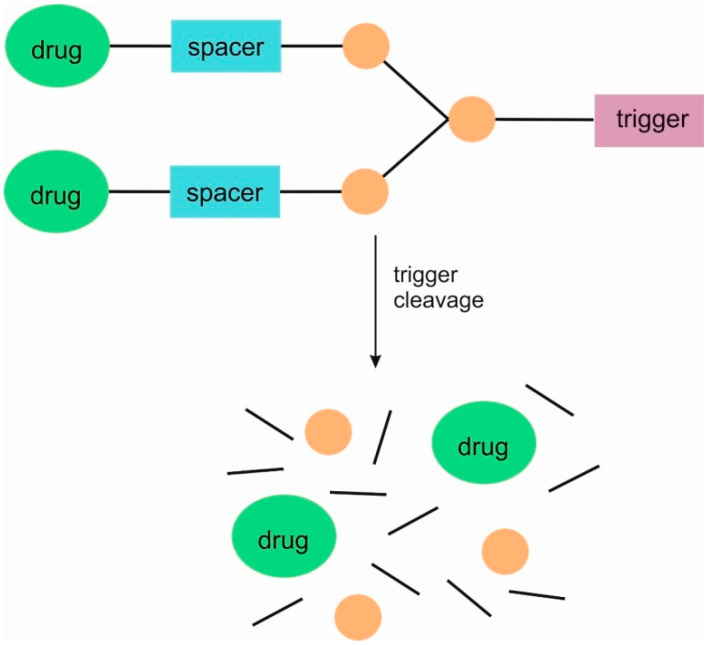
Structure of first-generation self-immolative domino dendrimers as anti-cancer drug delivery systems.

This type of dendrimer comprises a specifier/trigger, a spacer, and a drug. When drugs are covalently conjugated to dendrimers, these linkages must be designed to cleave chemically in order to release the active substance. Such linkages can be designed to cleave selectively at the therapeutic target under specific biological conditions [8]. SIDendr are designed such that cleavage of a trigger initiates a cascade of intramolecular chemical reactions, leading to the complete disintegration of the material into small molecule components (Figure 1). The first-generation dendrimer (G1) has one branching unit. 

The spacers are covalent assemblies tailored to correlate the cleavage of two chemical bonds in an inactive precursor. The precursor typically contains a protective capping moiety, the central spacer, and the compound of interest. After applying an appropriate stimulus (chemical, physical, or biological), the protective moiety is removed, generating a cascade of disassembling reactions, ultimately leading to the release of the active compound [10,11]. 

**Figure 2:**
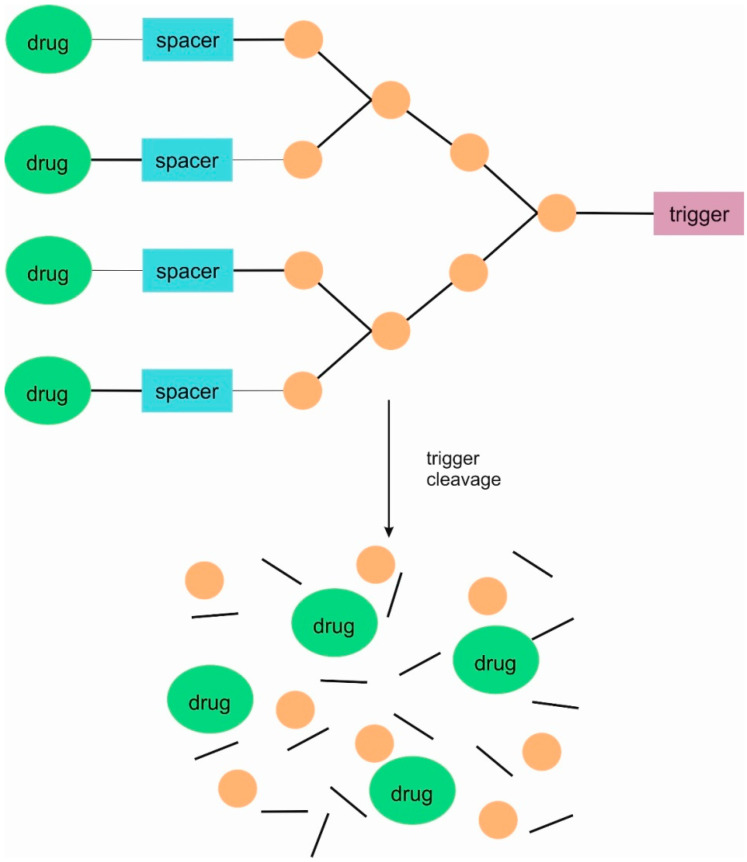
Structure of second-generation self-immolative domino dendrimers as anti-cancer drug delivery systems.

The higher-generation dendrimers are also known. There has been a further intensive development of subsequent generations of domino dendrimers as carriers of anti-cancer substances. The subsequent generations of domino dendrimers make it possible to obtain SIDendr-ac-DDS containing, among other things, a larger number of molecules of one or several anti-cancer drugs, fragments targeting cancer cells, or, in the future, imaging agents. This gives hope for the development of increasingly intelligent anti-cancer drug carriers that can increase oncological therapy’s effectiveness and biosafety. The second-generation dendrimer (G2) has two branching units (Figure 2), and the third-generation dendrimer (G3) has an additional three branching units.

**Figure 3:**
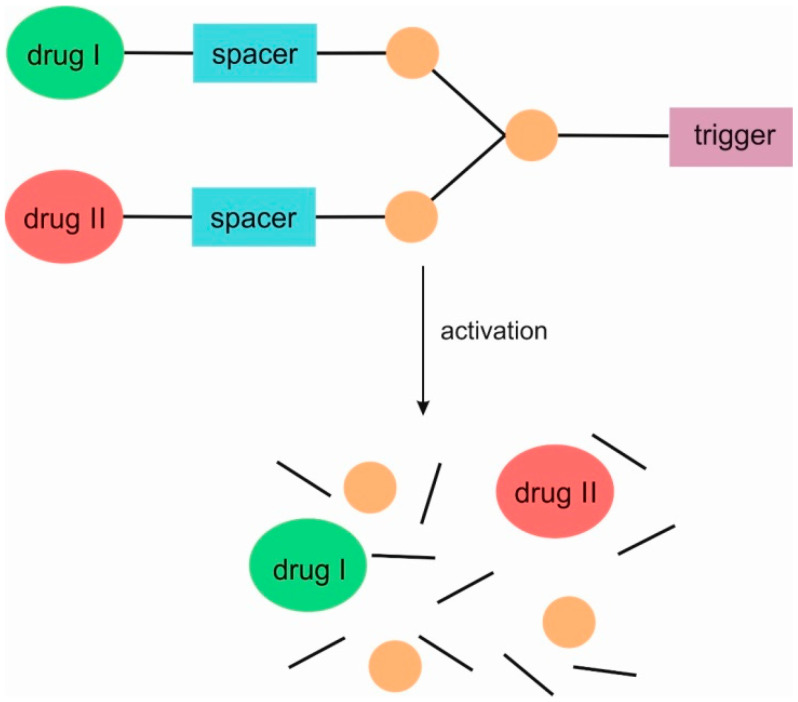
Structure of heterodimeric first-generation self-immolative domino dendrimers as anti-cancer drug delivery systems.

**Figure 4:**
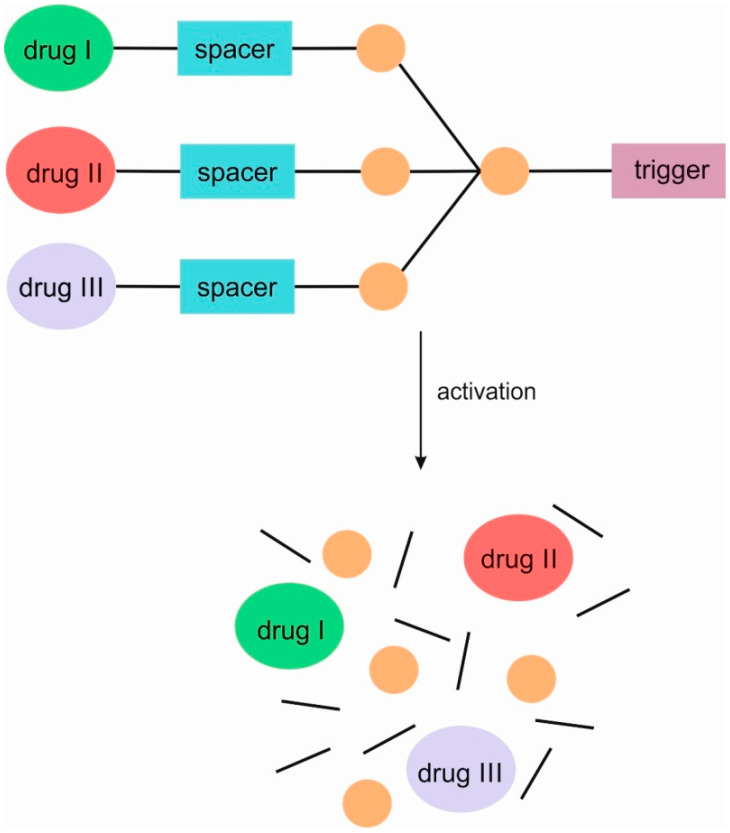
Structure of heterotrimeric first-generation self-immolative domino dendrimers as anti-cancer drug delivery systems.

SIDendr may contain one (homodimeric prodrugs) (Figure 1), two (heterodimeric prodrugs) (Figure 3), or three (heterotrimeric prodrugs) (Figure 4) drug molecules in their structure.

**Figure 5:**
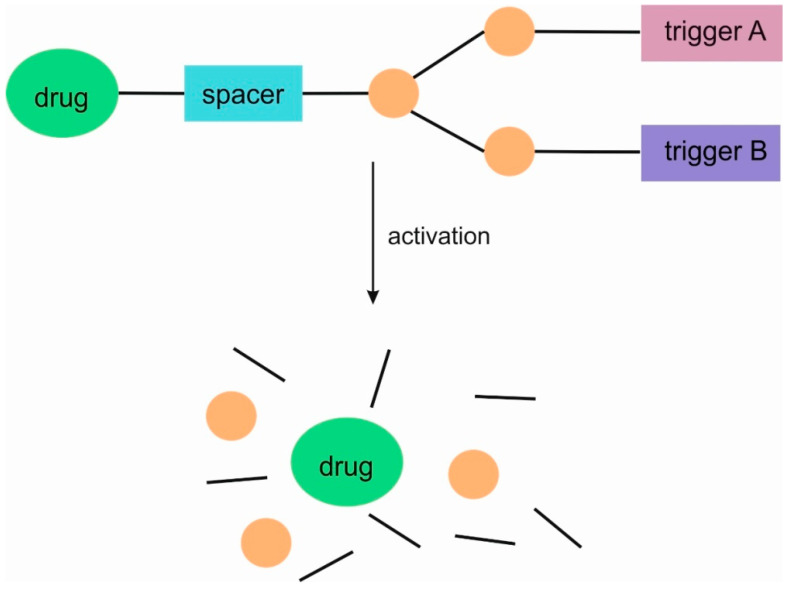
Structure of self-immolative domino dendrimers as anti-cancer drug delivery systems activated by two different triggers.

Furthermore, SIDendr can be activated by one (Figure 1) or two (Figure 5) types of triggers. The second type of SIDendr-ac-DDS contains two types of triggers in their structure compared to the system containing one type of trigger (Figure 1). The aforementioned system allows for the release of the drug, for example, in two time phases or a stronger drug release, due to the synergism of activation of two trigger types.

Numerous stimuli-responsive dendritic systems have been designed and synthesized up to now. In tumor sites, pH-responsive, reduction-responsive, oxidation-responsive, and enzyme-responsive SIDendr can respond to a lower pH (extracellular pH: ~6.8), a higher concentration of GSH (in the cytosol), enhanced intrinsic oxidative stress, and the elevated enzyme expression of tumor tissue, respectively.

At present, SIDendr have been developed with the following anti-cancer drugs: paclitaxel (PACL), camptothecin (CAMPT), doxorubicin (DOX), etoposide (ETOP), entinostat (MS-275), and melphalan (MELP) (Table 1).

## 2. First-Generation Self-Immolative Domino Dendrimers as Anti-Cancer Drug Delivery Systems

**Figure 6:**
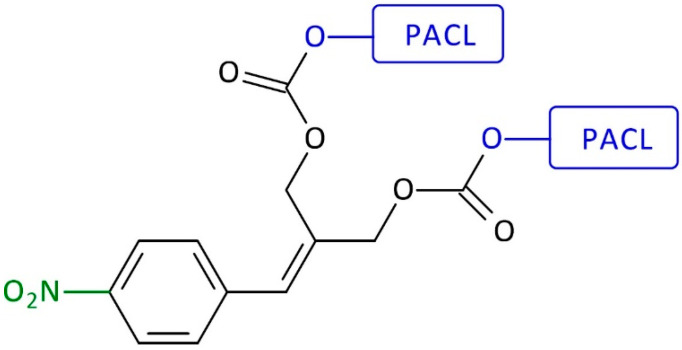
Structure of first-generation self-immolative domino-dendrimer-based paclitaxel delivery systems.

For the first time, the first-generation self-immolative dendrimer-based anti-cancer drug delivery systems (SIDendr-ac-DDSs) were obtained by de Groot et al. [20]. The dendritic molecular system was vinyl-benzylic moieties that could release terminal units via double 1,8-elimination. The nitro group was used to mask aniline at the focal point in its oxidized form. Two PACL molecules were conjugated to the rim of the SIDendr-ac-DDSs (Figure 6). The reduction of the nitro group to the amino group (with Zn catalyst, acetic acid conditions) launches a cascade of fragmentation via double 1,8-elimination, followed by decarboxylation reactions. All of those processes lead to the release of the terminal drug molecules from the dendrimer’s end unit. The undoubted advantage of the developed system is its straightforward structure and chemical activation in the presence of a non-toxic metal—zinc.

**Figure 7:**
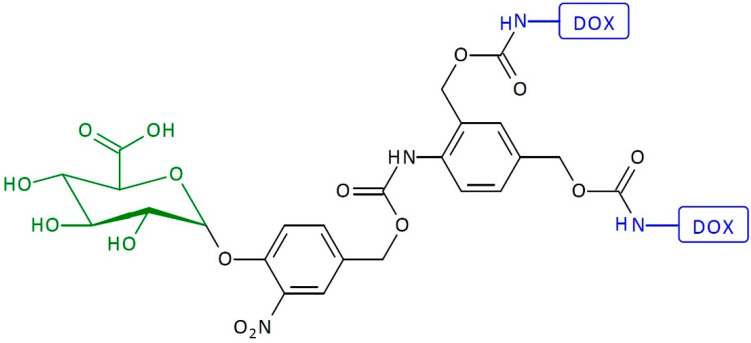
Structure of first-generation self-immolative domino-dendrimer-based doxorubicin delivery systems.

An example of an enzyme-activated SIDendr-ac-DDSs is a system containing a trigger substrate activated by β-glucuronidase and two DOX molecules (Figure 7) [21]. Initially, due to enzymatic activation, the hydrolysis of the dendrimer occurs. The sugar molecule splits off from the dendrimer. Next, the processes of 1,8-elimination and decarboxylation occur, releasing the drug molecule from the dendrimer. It was found that the dendritic system, upon β-glucuronidase activation, causes two times more toxicity toward H661 lung cancer cells than its monomeric counterpart. The advantage of this system is the relatively simple preparation method. Moreover, it is characterized by high toxicity towards cancer cells.

**Figure 8:**
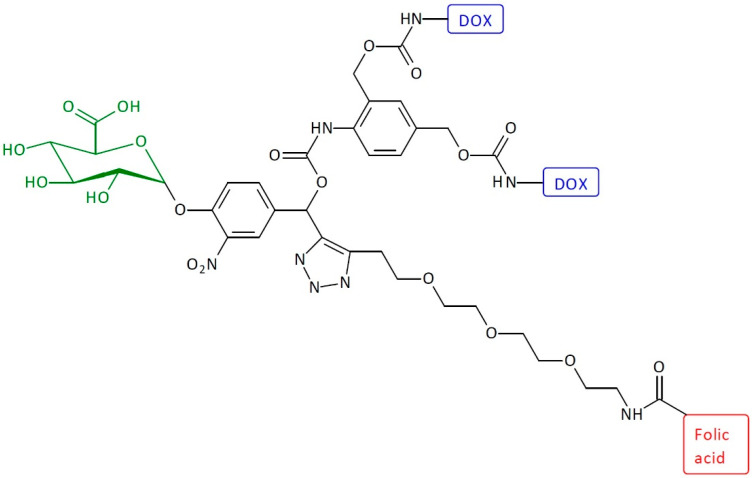
Structure of first-generation self-immolative domino-dendrimer-based doxorubicin and folic acid delivery systems.

A similar system was additionally conjugated with folic acid (FAc) (Figure 8) [22]. Grinda and coworkers showed a trigger with a lysosomal β-galactosidase-activated substrate incorporating FAc as a guide moiety. The obtained system comprises the following five units: a targeting ligand, an enzymatic trigger, a self-immolative spacer, and two active drugs articulated around a chemical amplifier. Two molecules of DOX are released after β-galactosidase activation and subsequent multiple elimination reactions. Because of the presence of the FAc, the SIDendr-ac-DDSs recognize a population of cells that autonomously express the folate receptor. The most significant advantage of the obtained system is the presence of a group in the structure that targets cancer cells. This significantly increases the pharmacological properties of the SIDendr-ac-DDS.

**Figure 9:**
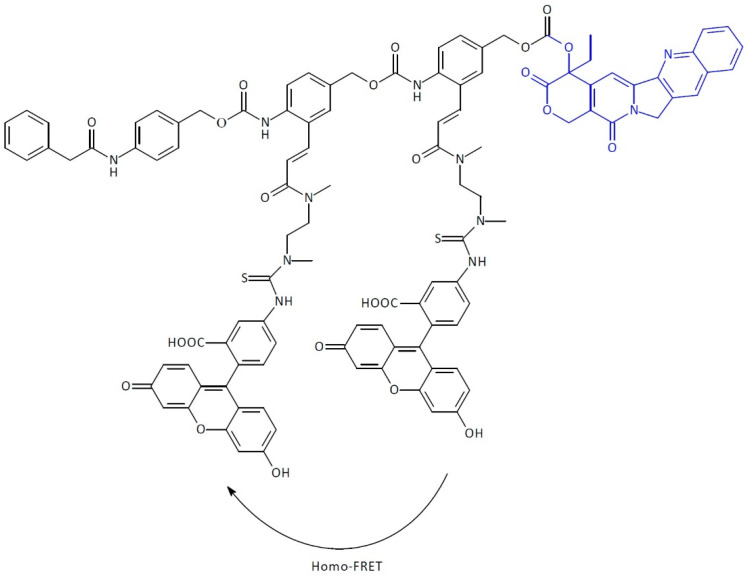
Structure of self-immolative domino-dendrimer-based camptothecin and dye delivery systems.

SIDendr-ac-DDSs were also developed for ‘theranostics,’ a combination of therapy and diagnostics. In the system, a turn-ON fluorescent diagnostic signal accompanies the disassembly of the prodrug and enables the monitoring of the active drug release. The signal is generated through the fragmentation of a dimeric self-immolative linker attached to a pair of FRET dyes. SIDendr-ac-DDS is one example of this, which relies on self-immolative dendritic units containing two molecules of dye located in close spatial proximity, thus extinguishing their fluorescence (Figure 9) [23]. SIDendr-ac-DDS is equipped with phenylacetamide as a penicillin-G-amidase (PGA) substrate and CAMPT coupled via a self-immolative bridge. The linker comprises two aniline adapter units attached to a pair of fluorescein dyes. Upon activation by the trigger group, the PGA exposes the aniline unit, resulting in sequential 1,6-eliminations that increase the distance between the released CAMPT and the fluorescent dyes. The obtained results demonstrate that an increase in the emitted fluorescence signal is observed after the specific activation of the prodrug. The most significant advantage of the obtained system is the fluorescent agent presence in the structure that allows the combination of oncology therapy and diagnostics. Moreover, the developed SIDendr-ac-DDSs were characterized by highly controlled drug release kinetics. The disadvantages include the relatively complicated SIDendr-ac-DDS synthesis method.

**Figure 10:**
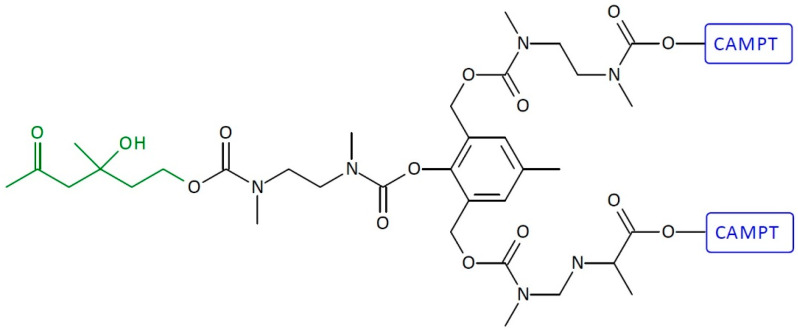
Structure of homodimeric first-generation self-immolative domino-dendrimer-based camptothecin delivery systems.

Other examples of homodimeric first-generation SIDendr-ac-DDSs are CAMPT- or DOX-containing systems (Figure 10) [24]. These anti-cancer medicine molecules were implanted with the retro-aldol, retro-Michael substrate of catalytic antibody 38C2 (CA 38C2) as a trigger. The retro-aldol, retro-Michael substrate of antibody 38C2 was attached to the adaptor platform through a self-immolative linker with adequate length to avoid steric hindrances accompanying the complex structure of the doxorubicin (DOX) molecule. The dendritic prodrug bioactivations were evaluated in a cell-growth inhibition assay with the Molt-3 leukemia cell line in the presence and the absence of mouse monoclonal antibody 38C2. The IC_50_ values of the monomeric prodrugs were about 200-fold less toxic than the free CPT. The activity of the monomeric prodrug had shifted to a 10-fold difference from that of the free CPT (upon the addition of 38C2). The main advantage of the obtained systems is the relatively simple preparation method and their very high activity towards cancer cells and low toxicity towards normal cells. 

**Figure 11:**
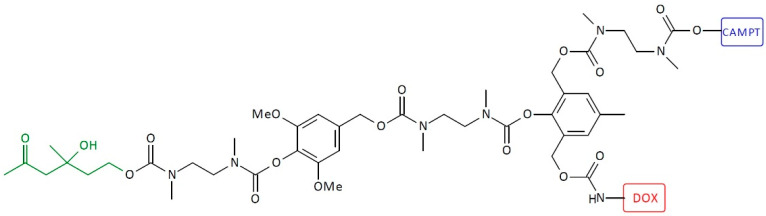
Structure of heterodimeric first-generation self-immolative domino-dendrimer-based camptothecin and doxorubicin delivery systems.

Heterodimeric first-generation SIDendr-ac-DDSs consisting of CAMPT and DOX as end units were also prepared (Figure 11) [24]. The IC_50_ values of the dimeric prodrugs were also about 200-fold less toxic than the free CAMP (like the monomeric prodrugs). However, the dimeric prodrug was about four times more active upon the addition of 38C2 in comparison to the monomeric prodrug. Moreover, a synergetic cytotoxic effect was noticed for such heterodimeric systems after bioactivation by CA 38C2. The aforementioned biological synergistic effect is an undoubted advantage of the developed systems. An additional positive is the simple method of synthesizing these SIDendr-ac-DDSs.

**Figure 12:**
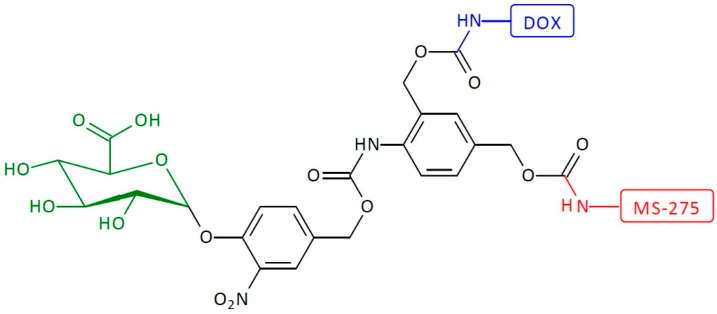
Structure of heterodimeric first-generation self-immolative domino-dendrimer-based entinostat and doxorubicin delivery systems.

Another example of heterodimeric first-generation SIDendr-ac-DDSs is a dendritic platform that works by 1,6- and 1,4-eliminations with a platform that includes the N,N′-dimethylethylenediamine moiety (Figure 12) [25]. The single-triggered SIDendr-ac-DDS consists of the β-glucuronidase enzymatic substrate, DOX, and histone deacetylase inhibitor (MS-275 units). The heterodimeric prodrug includes a nitrobenzylphenoxy carbamate linker between the glucuronide trigger and the amplifier unit. The enzyme substrate is located at a substantial distance from the two drug molecules, allowing easy recognition by β-glucuronidase. The enzyme-catalyzed cleavage of the prodrug results in the release of phenol intermediate, which induces the release of aniline through a 1,6-elimination process. Once turned on, the amplifier first causes the expulsion of DOX via a 1,4-elimination, followed by spontaneous decarboxylation. Adding water to ortho-azaquinone methide generates aniline, thereby permitting the release of MS-275. The most significant advantage of the developed systems is the incorporation of MS-275 into the SIDendr structure and their controlled release. As is known, histone deacetylase inhibitors are considered as new-generation anti-cancer drugs that induce increased histone acetylation. These compounds modulate the structure of chromatin, which leads to changes in the expression of many genes that influence signaling pathways, the inhibition of the cell cycle and angiogenesis, or the induction of apoptosis in cancer cells. Currently, many types of histone deacetylase inhibitors are at the same stage of clinical trials as monotherapy, or in combination with other cytostatics.

**Figure 13:**
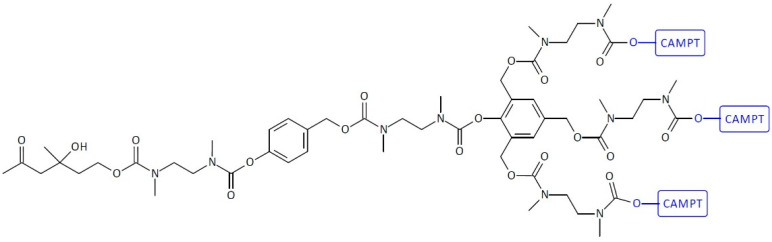
Structure of homotrimeric first-generation self-immolative domino-dendrimer-based camptothecin delivery systems.

A dendritic molecular system with three CAMPT molecules has been synthesized by Haba et al. (Figure 13) [26]. The authors synthesized the trimeric prodrug system in which three molecules of the anti-cancer drug CAMP (pro-CAMP) were linked through a retro-aldol, retro-Michael trigger to a substrate for the catalytic antibody 38C2. The trigger activation by CA 38C2 resulted in the re-leasing of three CAMPT molecules via a cyclization step and the triple elimination of quinone methide. The single-triggered homotrimeric SIDendr-ac-DDSs were stronger than the corresponding monomeric SIDendr-ac-DDSs mediated by the catalytic antibody 38C2. It was found that prodrugs in the presence of catalytic antibody 38C2 slowed down the cell proliferation for the following three different cell lines: the human T-lineage acute lymphoblastic leukemia (ALL) cell line MOLT-3, the human erythroleukemia cell line HEL, and the human acute myeloid leukemia (AML) cell line HL-60. The most significant advantage of the synthesized system is the enhanced cytotoxic effect on cancer cells compared to monomeric SIDendr-ac-DDSs.

**Figure 14:**
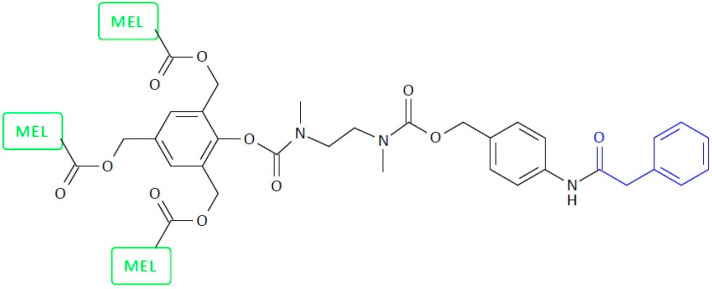
Self-immolative dendritic prodrug structure with tail units of melphalan and PGA-activated trigger.

Another example of a homotrimeric system is SIDendr-ac-DDSs with melphalan (MEL) tail units and a PGA-activated trigger (Figure 14) [27]. It was discovered that the trimeric prodrug system could offer outstanding benefits in the inhibition of tumor growth compared to common monomeric prodrugs (human T-lineage acute lymphoblastic leukemia MOLT-3 cells), mainly if the target or secreted enzyme (PGA) is present in relatively small amounts in the tumor tissue. It was found that MEL was released from the obtained systems with relatively high control. The main advantages of the synthesized systems are the enhanced cytotoxic effect on cancer cells compared to monomeric systems and the high control over the release of the anti-cancer drug from this SIDendr-ac-DDS.

**Figure 15:**
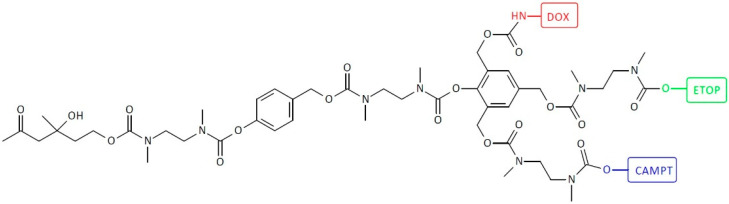
Structure of heterotrimeric first-generation self-immolative domino-dendrimer-based camptothecin, doxorubicin, and etoposide delivery systems.

Figure 15 depicts heterotrimeric SIDendr-ac-DDSs comprising CAMPT, DOX, and ETOP tail units and antibody-38C2-activated specific triggers [26]. Three different anti-cancer drugs are released almost simultaneously by a single cleavage event at the trigger site. The heterotrimeric prodrug system was evaluated in a cell-growth inhibition assay. The prodrug was incubated with MOLT-3 leukemia cells in the presence and absence of catalytic antibody 38C2. The inhibition of cell growth by the heterotrimeric prodrug was increased approximately 15-fold upon activation by antibody 38C2. A significant advantage of the synthesized SIDendr-ac-DDSs is the enhanced cytotoxic effect on cancer cells caused by the simultaneous release of the three anti-cancer drugs.

**Figure 16:**
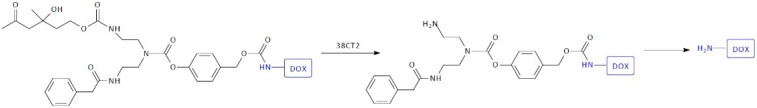
Structure of first-generation self-immolative domino-dendrimer-based doxorubicin activated through penicillin-G-amidase and catalytic antibody 38C2.

The dendrimer system containing two types of triggers is a fascinating concept. This device was named a novel prodrug system with activation gated through a molecular OR logic trigger [28] (Figure 16). The dendrimer has two different enzymatic substrates built into its structure, and DOX is used as an end unit. Phenylacetamides were chosen as the enzymatic substrates for PGA, and a retro-aldol, retro-Michael substrate is cleaved by CA 38C2. The cleavage of any of the triggers leads to DOX release and dendrimer degradation. The “OR” logic gate prodrug was assessed in cell growth inhibition assays. The authors evaluated the ability of the prodrugs to inhibit cell proliferation in the presence of PGA or catalytic antibody 38C2 using two different cell lines: the human T-lineage acute lymphoblastic leukemia cell line MOLT-3 and the human erythroleukemia cell line HEL. It was found that both PGA and antibody 38C2 activated the prodrug pro-DOX, and the cell growth was inhibited with IC_50_ values close to that of the parent drug. The most important advantages of the obtained SIDendr-ac-DDSs is the activation of the system by any trigger and a high cytotoxic effect on cancer cells. This makes it possible to activate the SIDendr-ac-DDSs in various biological stimuli and physiological conditions.

## 3. Second-Generation Self-Immolative Domino-Dendrimer-Based Anti-Cancer Drug Delivery Systems

**Figure 17:**
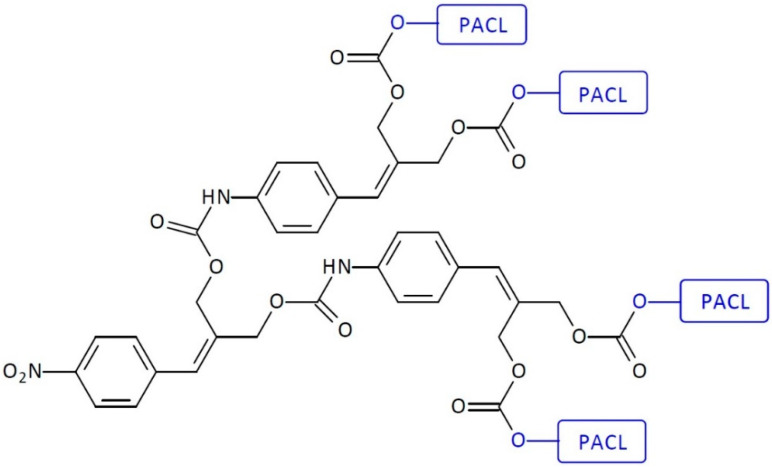
Structure of second-generation self-immolative domino-dendrimer-based paclitaxel delivery systems.

De Groot and coworkers reported the synthesis of a second-generation SIDendr-ac-DDS, achieved by the implantation of two additional molecules of 2-(4-aminobenzylidene)-propane-1,3-diol to the first-generation moiety. This dendrimer releases four PACL molecules (Figure 17) [20]. First, bis(4-nitrophenyl carbonate) was reacted with two molecules of aminodiol. Next, the obtained product was reacted with 4-nitrophenyl chloroformate. Finally, the product of this reaction was coupled to four equivalents of PACL to yield the second-generation, cascade-release dendrimer loaded with four PACL molecules at the dendritic termini. The most important advantage of the obtained systems is the achievement of an enhanced cytotoxic effect on cancer cells caused by the release of four anti-cancer drug molecules. A particular disadvantage of this solution is that the method of synthesizing the system is relatively complicated. 

## 4. Other Types of Self-Immolative Dendrimer-Based Anti-Cancer Drug Delivery Systems

**Figure 18:**
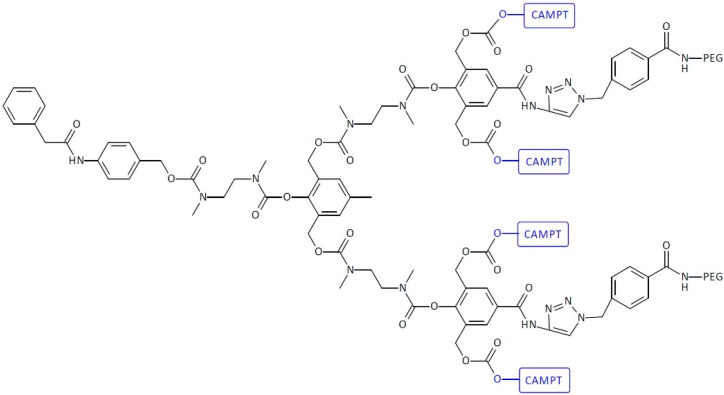
Structure of second-generation self-immolative dendrimers functionalized with poly(ethylene glycol)-based camptothecin delivery systems.

SIDendr-ac-DDSs with polymer unit fragments have also been created. The second-generation SIDendr-ac-DDSs have not been as promising as the first-generation ones, due to the hydrophobic-structure-caused aggregation, poor cleavage efficiency, and steric hindrance at the focal sites of the dendron. The dendrimer was grafted with poly(ethylene glycol) (PEG) to address the issue. The synthesis of the dendritic molecule was obtained in a number of steps. First, 4-hydroxybenzoic acid was coupled with propargylamine to produce amide, which was further reacted with formaldehyde. The obtained product was selectively protected with two equivalents of tert-butyl-dimethylsilyl chloride to generate a phenol derivative, which was then activated with p-nitrophenyl chloroformate to produce a carbonate derivative. The reaction of mono-Boc-N,N′-dimethylethylenediamine with the carbonate derivative afforded carbamate, which was stirred with amberlist in methanol to generate a compound with the blocking groups removed. Then, post-reactions were carried out with 4-nitrophenyl isocyanate, trifluoroacetic acid, and p-nitrophenyl chloroformate. The dendric compound was obtained. Finally, the dendritic molecule was conjugated with 2 equiv of PEG 400-azide via the copper-catalyzed click reaction to produce SIDendr-ac-DDSs with two PEG-5000 tails conjugated with four CAMPT molecules. The tails of the PEG molecules lowered the hydrophobic properties of the dendritic system and increased its aqueous solubility, thereby preventing aggregation. Gopin et al. obtained SIDendr-ac-DDSs with two PEG-5000 tails conjugated with four CAMPT molecules and a single-triggering substrate activated by PGA (Figure 18) [29]. It was found that PGA effectively activates the dendritic prodrugs, and its toxicity increased significantly in the cell-growth inhibition assays of three cancerous cell lines (the human T-lineage acute lymphoblastic leukemia cell line MOLT-3, the human leukemia T cell line JURKAT, and the human kidney embryonic HEK-293 cell line). It was also found that CAMPT was released from the dendrimer in the presence of PGA in a highly controlled manner over approximately 180 h. The main advantages of the obtained systems include the highly controlled release of the anti-cancer drug and a high cytotoxic effect on pathological cells. An undoubted disadvantage is the multi-step and complicated method of synthesizing this type of SIDendr-ac-DDS. 

**Figure 19:**
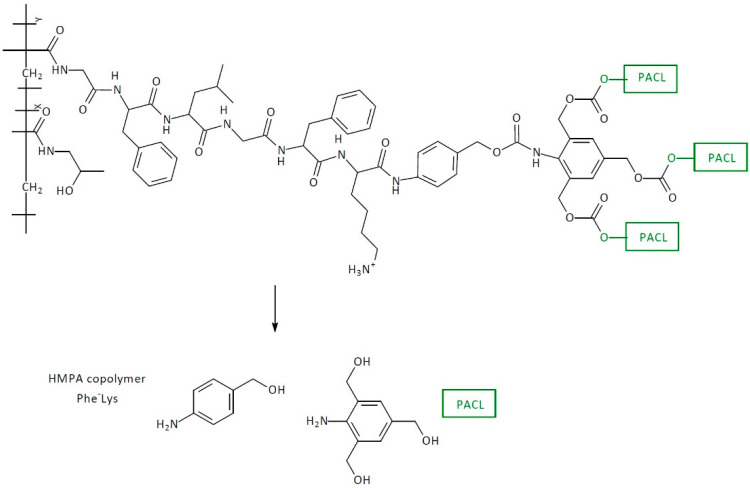
Structure of self-immolative dendrimers functionalized with poly(N-(2-hydroxypropyl)methacrylamide)-based paclitaxel delivery systems.

Another example is a dendric system conjugated with poly(N-(2-hydroxypropyl)methacrylamide) (pHPMA) (Figure 19) [30]. The pHPMA are ligands characterized by a high solubility in water, a lack of immunogenicity, and non-toxicity. The synthesis of DDS was carried out in a number of steps. First, the previously synthesized L-Boc-Phe-ONp25 was reacted with L-Lys(alloc)-OH to produce dipeptide. The latter was conjugated with 4-aminobenzyl alcohol to generate alcohol derivate. The isocyanate derivate was reacted with benzyl alcohol to produce carbamate, followed by deprotection with amberlyst to generate triol. The activation with 4-nitrophenyl chloroformate produced tricarbonate, which reacted with three equivalents of PACL to yield a dendric compound. Next, the deprotection of the terminal Boc group with TFA allowed the conjugation with pHPMA copolymer-Gly-Phe-Leu-Gly-ONp. The remaining activated sites of the copolymer were conjugated with excess aminoethanol to generate a compound, which, after deprotection of the Lys residue, afforded the desired SIDendr-ac-DDS. In this way, they enable targeted drug delivery to tumor cells, thanks to the improved effects of permeability and retention (EPR). This type of SIDendr-ac-DDS releases a triple charge of the hydrophobic PACL after the cleavage of the Gly-Phe-Leu-Gly substrate by the endogenous enzyme cathepsin B. The bioconjugate pHPMA–dendritic PTX showed higher cytotoxicity against murine prostate adenocarcinoma cells compared to the monomeric pHPMA–PACL. The undoubted advantages of the obtained systems include the highly controlled release of anti-cancer drugs and the high cytotoxic effect on cancer cells. The disadvantage is the multi-step and highly complex method of synthesizing this type of SIDendr-ac-DDS.

**Figure 20:**
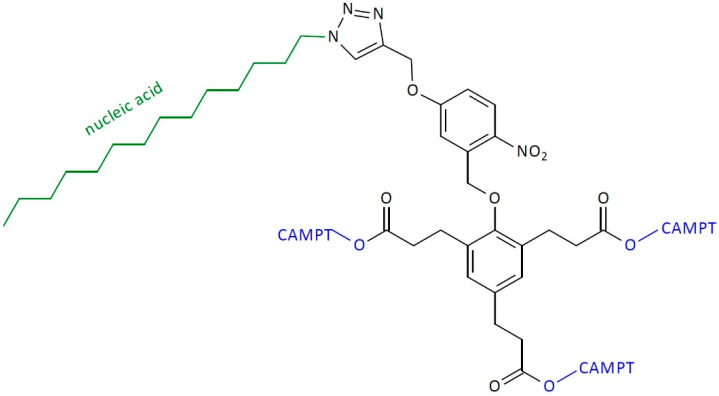
Structure of self-immolative dendrimers functionalized with nucleic-acid-based camptothecin delivery systems.

An exciting example of this is the use of nucleic acid to obtain SIDendr-ac-DDSs (Figure 20) [31]. Tan and coworkers synthesized the UV-responsive SIDendr-ac-DDSs built on a phenol dendric platform linked through carbonate bonds to three CAMPT molecules at the end sites. The DNA was conjugated to the dendrimer molecule using the “click reaction” method. The coupling process of the dendric compound and azide-modified DNA was carried out in the presence of CuBr and tris(benzyltriazolylmethyl)amine. The amphiphilic prodrug was prepared by conjugating the SIDendr-ac-DDSs to a DNA strand. It was observed that, in an aqueous environment, the SIDendr-ac-DDSs could self-organize into nanostructures. The nanostructures were rapidly taken into cells, and the attached DNA was resistant to nuclease cleavage. The effect of UV irradiation at 365 nm caused the separation of the nucleic acid shell. Thus, the exposition of phenolate of the self-immolative dendron and the subsequent triple elimination released the CAMPT. It was found that the DNA–CAMP exhibited much reduced toxicity compared with the free CPT in the absence of UV light. Furthermore, adding UV irradiation did not increase the cytotoxicity of the free CAMP. Moreover, the cell-killing efficacy of the DNA−CAMP conjugate was significantly amplified by the UV treatment [31]. The most important advantage of the obtained SIDendr-ac-DDSs is their easy penetration into cancer cells and the highly controlled system activation. A certain disadvantage is the relatively complicated synthesis method.

**Figure 21:**
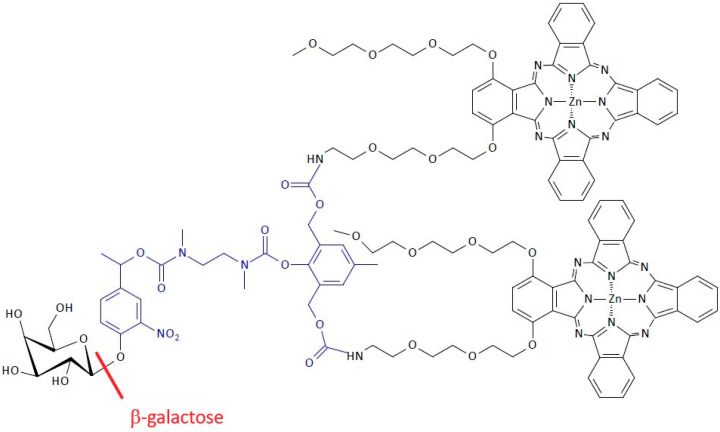
Structure of β-gal-activatable nanophotosensitizing system for the detection and elimination of senescent cells.

Chu et al. have developed an interesting nanophotosensitizing approach of SIDendr with a high potential for use in cancer therapy (Figure 21) [32]. This novel system responds to the senescence-associated β-galactosidase (β-gal) to detect and eliminate cancer cells selectively. A significant advantage of the obtained SIDendr-ac-DDSs is the high selectivity and destruction of pathological cells. Unfortunately, a particular disadvantage is this system’s very complicated synthesis method.

It involves a dimeric zinc(II) phthalocyanine (ZnPc) linked to a β-gal unit via a self-immolative linker. The β-gal-activatable Gal-(ZnPc)_2_ was prepared by the condensation of ZnPc and the β-galactose-substituted AB_2_-type self-immolative linker in N,N-dimethylformamide (DMF), followed by the hydrolysis of the intermediate product to remove the acetyl groups. The Gal-(ZnPc)_2_ compound in an aqueous environment that can self-assemble, forming stable nanoscale particles in which the phthalocyanine units are stacked and self-quenching to emit fluorescence and produce singlet oxygen. Once internalized into senescent HeLa cells, these nanoparticles interrelate with the senescence-associated, overproduced β-gal inside of the cells to trigger the disassembly process through the enzymatic cleavage of the glycosidic bonds. This is followed by self-immolation to release the photoactive monomeric phthalocyanine moieties. Fluorescence can light up these senescent cells and eliminate them through photodynamic action when irradiated with light [32]. The nanosystem can serve as an efficient fluorescent probe for detecting cellular senescence, due to the 4.5-fold higher intensity of the fluorescence in the senescent cells compared to the proliferating cells. It is also possible to activate the intracellular ROS generation ability, enabling the effective killing of the senescent cells with an IC_50_ value as low as 0.06 μM [32].

**Figure 22:**
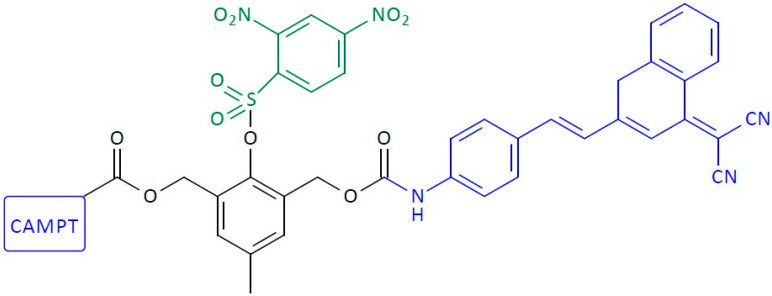
Structure of self-immolative dendrimer-based camptothecin and dicyanomethylene-4H-pyran delivery systems.

More interesting SIDendr for in situ drug release tracking and anti-cancer treatment have been synthesized (Figure 22) [33,34]. The self-immolative SIDendr comprise the reporter groups, the anti-cancer drug (CAMPT), a two-photon NIR fluorophore (dicyanomethylene-4H-pyran, DCM), and the glutathione (GSH)-trigger. The core of the dendrimer compound was obtained by linking 2,4-dinitrobenzenesulfonylchl to 2,6-bis(hydroxymethyl)-4-cresol through a sulfonate linkage. Next, one hydroxyl was protected using t-butyldimethylchlorosilane, allowing the remaining hydroxyl to link with a NIR dye DCM (through a carbamate bond) to produce a semi-finished product. After the deprotection of the hydroxyl, an anti-cancer drug, CAMPT, was linked to the structure (through a carbonate bond) to yield SIDendr-ac-DDSs. A significant and unique advantage of the synthesized SIDendr-ac-DDSs is the ability to observe cancer cells in various phases of their life cycle and the effect of their elimination.

GSH is an endogenous tripeptide in cells at millimolar concentrations and is a major antioxidant in many biological systems. The dendritic-drug-releasing system contains 2,4-dinitrobenzenesulfonyl (DNS) as the thiol-responsive trigger group, which was cleaved by GSH treatment to release the corresponding phenol. This facilitated self-immolation by combining 1,4-elimination processes to supply CAMPT and DCM to cellular environments. It was proved that this prodrug is effective both in vitro and in vivo. It enables CAMPT sustained release and effectively inhibits tumor growth [33,34].

Zeng and Wu also prepared similar SIDendr containing two CAMPT molecules [33,35]. After the reduction of the 1,4-benzoquinone to the corresponding hydroquinone, the subsequent cyclization of the trimethyl-lock and N,N′-ethylenediamine linkers occurs. This is followed by the 1,4-elimination of the phenol unit and decarboxylation to CAMPT reporter group release. The fluorescence resulting from CAMPT irradiation is quenched by the quinone. However, the dendron fragmentation liberates the topoisomerase I inhibitor and re-establishes CAMPT fluorescence (λ_max_ 446 nm), self-indicating fragmentation and quantifying DT-diaphorase activity. The authors showed that release occurs in A549 cells (a DT-diaphorase overexpressing cell line) but not in L929 cells (a non-overexpressing cell line) [35].

**Figure 23:**
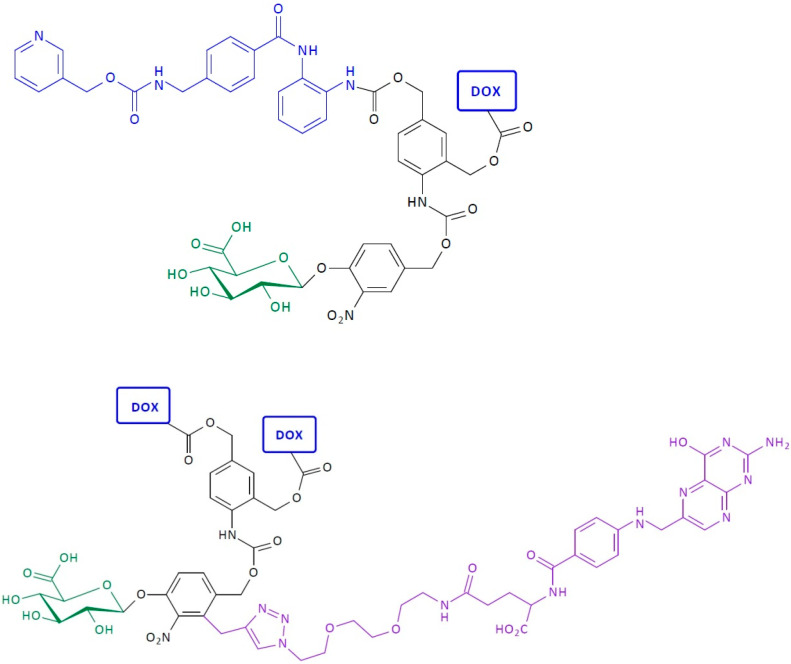
Structure of self-immolative dendrimer-based doxorubicin and delivery systems.

Another two types of interesting SIDendr containing one or two DOX molecules have been obtained by Papot and co-workers (Figure 23) [36]. The general pathway for preparing prodrugs containing an ortho-substituted phenyl ring involved the initial coupling of the methyl(2,3,4-tri-O-acetyl-R-D-glucopyranosyl bromide)uronate easily prepared from the D-glucuronolactone by ring opening with MeONa, then peracetylation and bromination (HBr/AcOH)] with the phenol derivatives in the presence of silver oxide. Coupling with DOX led to various glycosides. The deprotection process successively produced the fully deprotected prodrugs. The prepared SIDendr-ac-DDSs exhibited a strongly reduced cytotoxicity against the murine L1210 cell line compared to DOX. They remained almost 100-fold less toxic than DOX. Moreover, all of the SIDendr-ac-DDSs were highly stable in plasma. The most important advantages of the above SIDendr-ac-DDSs include very high cytotoxic activity towards cancer cells and their high stability in the biological environment. However, the disadvantage is the multi-stage and complicated preparation method for these SIDendr-ac-DDSs. 

The first SIDendr are phenol-based and attached to the glucuronic trigger group. The second are branched aniline-based enhancer units containing two distinct groups of reporters. The first featured DOX and the histone deacetylase inhibitor MS-275 as the reporter units. The ortho-nitro substituent included in the phenol-based linker assists in decreasing the pK_a_ of the phenol. It may influence the Michaelis–Menten constant of the substrate for the enzyme. This linker ensures an appropriate distance between the enzyme–substrate and the two bulky drugs, allowing for easy prodrug identification by β-glucuronidase. The enzyme-catalyzed prodrug cleavage caused phenoxide intermediate creation, which induced the aniline liberation in the 1,6-elimination process. Upon activation, the aniline enhancer unit first released DOX via 1,4-elimination and then spontaneous decarboxylation. The reaction with water in an aqueous solution traps the resulting ortho-azaquinone methide to regenerate the electron-rich aniline, allowing the subsequent liberation of MS-275 via the 1,6-elimination process. It is generally observed that 1,6 eliminations are faster than their 1,4 counterparts. However, this appears to be affected mainly by the nature of the leaving group [33,36].

The second SIDendr contain a folate unit that targets the folate receptors, often overexpressed in cancer cell lines, and facilitate prodrug endocytosis. The phenoxy linker structure was modified at the benzylic position to enable folate attachment via ‘click’ chemistry. The lysosomal enzyme β-galactosidase triggered self-immolation to liberate two DOX molecules in the most targeted manner [33,36].

**Figure 24:**
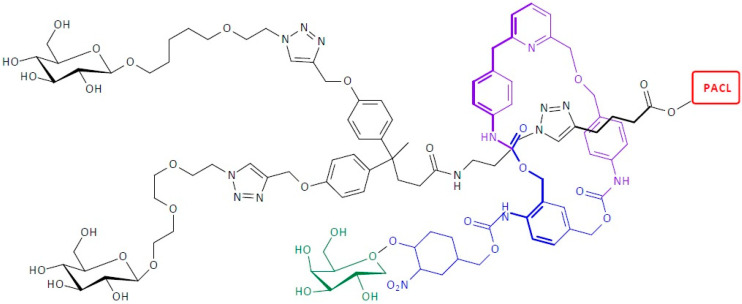
Structure of [2]-rotaxane self-immolative dendrimer-based paclitaxel delivery systems.

Supramolecular SIDendr based on [2]-rotaxane were also obtained. A PACL-prodrug, with an esterase-cleavable linker, is mechanically linked to the macrocyclic ring (Figure 24) [33,37]. The crucial step in the synthesis of the DDS relies on constructing the interlocked architecture via the Cu(I)-catalyzed azidealkyne 1,3-cycloaddition active-metal template strategy. The coordination of Cu(I) to the endotopic pyridine-containing macrocycle allows the azide and alkyne to bind to the metal catalyst in such a way that the formation of the triazole thread occurs predominantly within the cavity of the macrocycle, leading to the rotaxane architecture. The galactosylated ring was prepared from biscarbonate and dianiline derivatives. The macrocycle was treated with azide, alkyne, and Cu(CH_3_CN)_4_PF_6_ for 45 h at room temperature in CH_2_Cl_2_/CH_3_OH (87/13) to afford [2]-rotaxane. β-galactosidase triggers a self-immolative linker, which is built in the macrocyclic ring. The initiation of the first phenol-based linker takes place upon exposure to the enzyme via 1,6-elimination. In the next step, the 2,4-substituted aniline undergoes 1,6- or 1,4-elimination to fragment the locking ring, exposing the prodrug cleavable bond. The antiproliferative activity of the DDSs on HUVEC normal cells was evaluated. Compared to the cancer cells tested in this study, the DDS was less toxic for the HUVEC cells, with only a 20% reduction in cell viability at the highest tested dose [33,37]. The most important advantage of the developed system is the lack of toxicity towards normal cells, which allows for the selective destruction of cancer cells. A significant disadvantage is the very complicated and multi-step synthesis of this SIDendr-ac-DDS.

**Figure 25:**
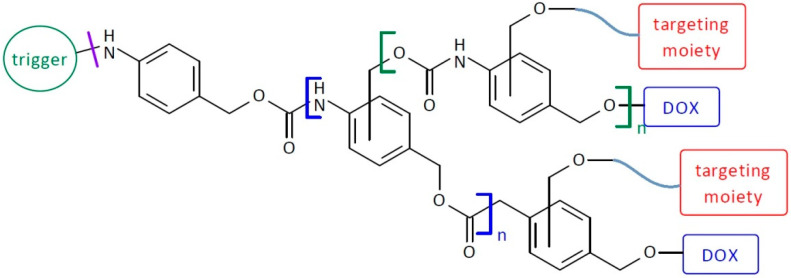
Structure of self-immolative dendrimer-based doxorubicin delivery systems.

Hyperbranched SIDendr with a poly(benzyl-carbamate) backbone were synthesized by Liu et al. (Figure 25) [38]. 

The monomeric unit of this polymer consists of a building block that is applied to synthetize the SIDendr. The reaction of polycondensation between hydroxybenzyl substituents and isocyanates of the monomeric building block produces the expected hyperbranched poly(benzyl-carbamate) polymer, which is then crowned with various substrates as primary triggers (responsive group for hydrogen peroxide or thiols). Fluorescent dyes (fluorescent group amino-coumarin) or DOX molecules are incorporated at the dendritic end sites. HS-cRGD (integrin targeting) and CGKRK (mitochondrion targeting) are used to target the moiety groups. The exposure of these molecules to a specialized analyte initializes the domino-like degradation of the hyperbranched polymer through the sequential eliminations of quinone-methide. As a result, a single-head trigger cleavage event results in the liberation of multiple end groups. In terms of structure, the molecular configuration of this type of polymer consists of a composition of SIDendr molecular structures and self-immolative polymers [38]. The most important advantage of the developed system is the integration of groups into the structure of the SIDendr-ac-DDSs that target cancer cells and the possibility of their precise elimination. The main disadvantage of this solution is the complicated synthesis method.

Zhang et al. created SIDendr-based stimuli-responsive drug-conjugated peptides for cancer treatment [39]. They applied lysine peptide conjugated with PEG and gemcitabine (dendrimer-GEM) with nanoparticles by click reaction. It was found that the produced nanoparticles with the glycyl phenylalanyl leucyl glycine tetra-peptide (GFLG) acting as a cleavable linker sensitive to enzymes are able to release the drug much faster in the tumor cellular environments, which contain explicitly secreted cathepsin B. It was presented that nearly 90% of GEM was released in the presence of the enzyme compared to the absence of cathepsin B. Such enzyme-sensitive systems provide benefits through their specific functions in biological and metabolic pathways [39].

Wang et al. prepared a drug delivery system based on SIDendr-CAMPT-based hydrogel with low cross-linking density [40]. In this type of DDS, a pH-controlled self-cleaving release of drug is accomplished via the ammonolysis of the ester bonds between the CAMPT and the dendrimer. The controlled self-cleaving liberation mechanism significantly prolongs the CAMPT release compared to the physically embedded drugs, thus enhancing tumor inhibition. The CAMPT is modified so that the end group is acrylate and, after that, is grafted onto the dendrimer surface. The dendrimer–drug conjugates are then reacted with PEG-DA to produce a weakly cross-linked DH (DH-G3-CPT). This DH drug delivery system with self-cleaving CAMPT was found to have sustained drug release compared to the physically embedded CAMPT. The DH-G3-CAMPT showed excellent tumor suppressive effects when intratumorally injected in a head and neck cancer model [40].

**Figure 26:**
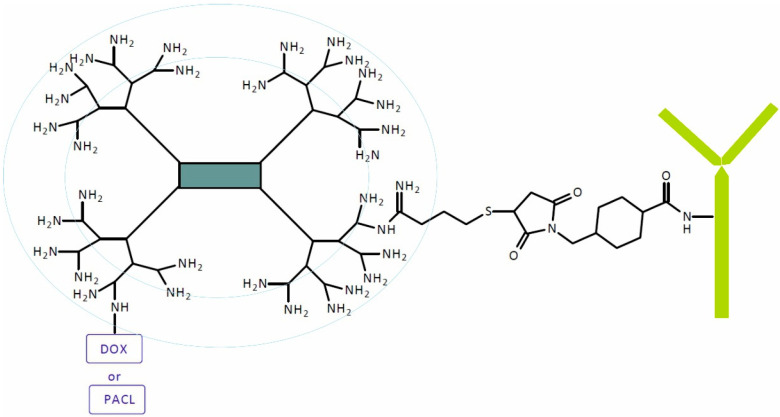
Structure of self-immolative dendrimer-based paclitaxel or doxorubicin delivery systems.

Two SIDendr trastuzumab (TMAB) and DOX or PACL specifically targeted to cells that overexpressed human epidermal growth factor receptor 2 (HER-2) were obtained by Marcinkowska and coworkers (Figure 26). Drugs and PAMAM (G4) were conjugated in the presence N-(3-dimethylaminopropyl)-N′-ethylcarbodiimide hydrochloride (EDC). The PAMAM–drug–TMAB conjugate was obtained in the reaction of derivatized TMAB and thiolated PAMAM–drug conjugate at a 1:12 molar ratio [41]. 

It was found that the SIDendr, in particular, showed extremely high toxicity toward the HER-2-positive SKBR-3 cells and very low toxicity towards to the HER-2-negative MCF-7 cells. As expected, the HER-2-positive SKBR-3 cell line accumulated TMAB from both conjugates rapidly. However, a large amount of PAMAM–PACL–TMAB conjugate was observed in the HER-2-negative MCF-7 cells. Confocal microscopy confirmed the intracellular localization of the analyzed compounds. The critical result of fluorescent imaging was the identification of the strong selective binding of the PAMAM–DOX–TMAB conjugate with HER-2-positive SKBR-3 cells only. The observed selectivity is achieved not only by including TMAB, which binds and blocks HER-2, but also by selecting a pH-sensitive linker that breaks in the tumor environment to allow PAMAM–drug conjugate release. Both of the conjugates show potential as DDSs, enhancing the therapeutic index and reducing the required dosage of anti-cancer drugs [41].

Ma et al. obtained the antibody–SIDendr for the targeting and release of chemotherapeutics at the tumor site while reducing the unwanted side effects due to drug accumulation in healthy tissues. DDSs were obtained by synthesizing the following conjugates: PAMAM–PTX, PEG–PAMAM–PTX, and TMAB–PEG–PAMAM–PTX [42]. They used TMAB, which binds to HER2, as a targeting agent in a TMAB–PAMAM conjugate transporting PACL specifically to HER2-overexpressing cells. They characterized the HER2-targeting TMAB–PEG–PAMAM–PTX conjugate as spherical particles with a narrow size distribution. The cells and tumors that overexpress HER2 can be specifically targeted by these SIDendr. The in vitro cytotoxicity of the TMAB-functionalized PAMAM–PACL conjugate was lower than that of the free PACL. However, it was beneficial in ameliorating the side effects when used in vivo, due to its excellent ability to target tumor tissues [42]. The main advantage of the above SIDendr-ac-DDSs is their extremely high toxicity towards cancer cells and their highly controlled release of anti-cancer drugs. The undisputed disadvantage is the complicated synthesis method of this type of SIDendr-ac-DDS.

## 5. Conclusions and Perspectives

SIDendr offer various biomedical applications due to their functional and structural versatility. They can also be employed as anti-cancer drug delivery systems. This work demonstrates that SIDendr structures with multiple advantages can suffer modifications to ensure anti-cancer drug transport and targeted delivery. The many already synthesized dendrimers, when used as carriers for anti-cancer drugs, should cause medical breakthroughs, including personalized medicine. However, this is not currently the case. The toxicity of various SIDendr poses a constraint to their usage in cancer therapy, prompting the development of various toxicity reduction strategies. In addition, we have noticed an absence of comprehensive information from biological studies conducted on both cellular and animal models. The promise of increasing the amount of this type of dendrimer employed in human health lies in recent preclinical and planned studies. 

In conclusion, the main advantages of SIDendr-ac-DDSs include the following:

- The possibility of cascading fragmentation of the dendrimer system into small, non-toxic fragments and molecules with the simultaneous release of the anti-cancer substance molecule(s);
- The fragmentation of dendrimers can be activated by various physical, chemical, and biological stimuli (external and internal);
- The sizes of the SIDendr-ac-DDSs (nanoscale) enable active or passive transport in the body and the precise targeting of cancer cells;
- The possibility of the very selective action of DDSs (the system can be activated only by one specific stimulus under optimal environmental conditions);
- The possibility of incorporating molecular targeting groups into the SIDendr-ac-DDS structure;
- The ability to activate the SIDendr-ac-DDSs inside of the cells or in the intercellular space;
- The possibility of obtaining systems containing molecules of two or three different anti-cancer substances (potential multi-drug therapy);
- The possibility of incorporating various types of triggers into the structure of the SIDendr-ac-DDSs (the possibility of releasing two or three different anti-cancer substances with different kinetics or releasing one anti-cancer substance in two phases);
- The high control of the kinetics of the release of anti-cancer substances;
- The possibility of obtaining SIDendr-ac-DDSs containing a different number of molecules of anti-cancer substances (or two/three types of anti-cancer substances), characterized by the expected kinetics of their release (potential personalized therapy).
- The main disadvantages of SIDendr-ac-DDSs are as follows:
- The relatively complicated synthesis methods (including many stages, prolonged reaction time, the low efficiency of some stages, multi-stage processes for purification of intermediates and products, etc.);
- The high cost of obtaining SIDendr-ac-DDSs (high cost of substrates, catalysts, and some solvents, as well as multi-stage processes);
- The incomplete knowledge about scaling the process;
- The incomplete knowledge about the pharmacological effectiveness and biosafety of SIDendr-ac-DDSs (lack of clinical trials).

Thus, there are still many pending works, but SIDendr can be considered an emerging opportunity in oncology and, in part, in personalized medicine.

## Figures and Tables

**Table 1:** Characterization of anti-cancer drugs applied to the development of self-immolative domino-dendrimer-based anti-cancer drug delivery systems [12,13,14,15,16,17,18,19].

Drug	General Characteristics	Application in Oncological Therapy
Doxorubicin (DOX)	The compound is a 14-hydroxylated version of daunorubicin, the direct precursor of DOX in its biosynthetic pathway. It interacts with DNA by intercalation and the inhibition of macromolecular biosynthesis. This inhibits the progression of topoisomerase II, an enzyme that relaxes the supercoils in DNA for transcription. It stabilizes the topoisomerase II complex after it has broken the DNA chain for replication, preventing the release of the DNA double helix and thus stopping the replication process. It may also increase the production of the quinone-type free radical, thereby contributing to its cytotoxicity.	Breast cancer, bladder cancer, stomach cancer, lung cancer, ovarian cancer, multiple myeloma, Kaposi’s sarcoma, lymphoma, and acute lymphocytic leukemia.
Paclitaxel (PACL)	A compound from the group of terpene alkaloids of the taxane type with a cytostatic effect. It is a secondary metabolite produced by *Taxus* sp., Coniferales Cephalotaxus, Podocarpus gracilior, or Corylus avellana. It is a phase-specific drug (G2 phase and M phase). Its antimitotic effect is based on the inhibition of microtubule depolymerization, which prevents, during cell division, the sister chromatids’ correct separation and the sister chromosomes’ migration.	Breast cancer, ovarian cancer, lung cancer, head and neck cancers, and Kaposi’s sarcoma.
Camptothecin (CAMPT)	It is a cytotoxic alkaloid found in indigenous trees in China and Tibet that contributes to the plant’s herbicidal defense mechanisms. It inhibits the replication of cancer cells by interacting with DNA topoisomerase 1. CAMPT prefers the inhibition of the Top1–DNA complex over the inhibition of the free topoisomerase1 enzyme. This enzyme expression in cancer cells is much higher than that seen in healthy cells, which allows for targeted selectivity.	Lung, ovarian, breast, pancreas, and stomach cancer.
Etoposide (ETOP)	Etoposide is a podophyllotoxin semisynthetic derivative from the rhizome of the wild mandrake (Podophyllum peltatum). More specifically, it is a glycoside of podophyllotoxin with a D-glucose derivative. It is a phase-specific drug (acts in interphase). The exact mechanism of action is not known, but it is known that etoposide works by the following mechanisms: breaking of single- and double-stranded DNA, the inhibition of topoisomerase II, the formation of free radicals, and the inhibition of thymidine incorporation into DNA.	Testicular cancer, lung cancer, lymphoma, leukemia, neuroblastoma, and ovarian cancer.
Entinostat (MS-275)	A characteristic synthetic benzamide derivative that belongs to class I of histone deacetylase inhibitors.	Breast cancer, non-small cellular lung cancer, Hodgkin lymphoma, colon cancer, and pancreatic cancer.
Melphalan (MELP)	It chemically changes, through alkylation, the DNA nucleotide guanine, which causes bonds between the DNA strands. This chemical change inhibits DNA and RNA synthesis, functions that cells need to survive. These changes cause cytotoxicity in both dividing and non-dividing cancer cells.	Multiple myeloma, ovarian cancer, melanoma, and primary amyloidosis.

## Abbreviations

CA 38C2	catalytic antibody 38C2
CAMPT	camptothecin
DCM	dicyanomethylene-4H-pyran
DDS, DDSs	drug delivery system(s)
DNS	2,4-dinitrobenzenesulfonyl
DOX	doxorubicin
EDC	N-(3-Dimethylaminopropyl)-N-ethylcarbodiimide hydrochloride
ETOP	etoposide
EPR	enhanced permeability and retention effect
Fac	folic acid
G1	first-generation dendrimer
G2	second-generation dendrimer
G3	third-generation dendrimer
β-gal	β-galactosidase
GSH	glutathione
HER2	human epidermal growth factor receptor 2
pHPMA	poly(*N*-(2-hydroxypropyl)methacrylamide)
MELP	melphalan
MS-275	entinostat
PACL	paclitaxel
PAMAM	polyamidoamine
PEG	polyethylene glycol
PGA	penicillin-G-amidase
SIDendr	self-immolative domino dendrimers
SIDendr-ac-DDS(s)	self-immolative dendromer-based anti-cancer drug delivery system(s)
TMAB	trastuzumab
Top1	topoisomerase 1ZnPc—dimeric zinc(II) phthalocyanine
